# Supplementary material for: Alloferon Affects the Chemosensitivity of Pancreatic Cancer by Regulating the Expression of SLC6A14
Source: Biomedicines. 2022 May 11;10(5):1113. doi: 10.3390/biomedicines10051113 (PMC9138528; doi:10.3390/biomedicines10051113)
Supplement: Supplementary file 1 [file biomedicines-10-01113-s001.zip › biomedicines-supplementary.pdf]

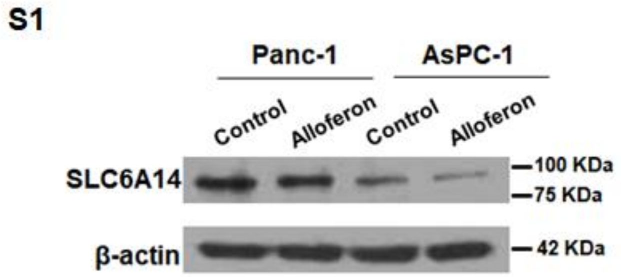

Figure S1: Difference of SLC6A14 expression between Panc-1 and AsPC-1

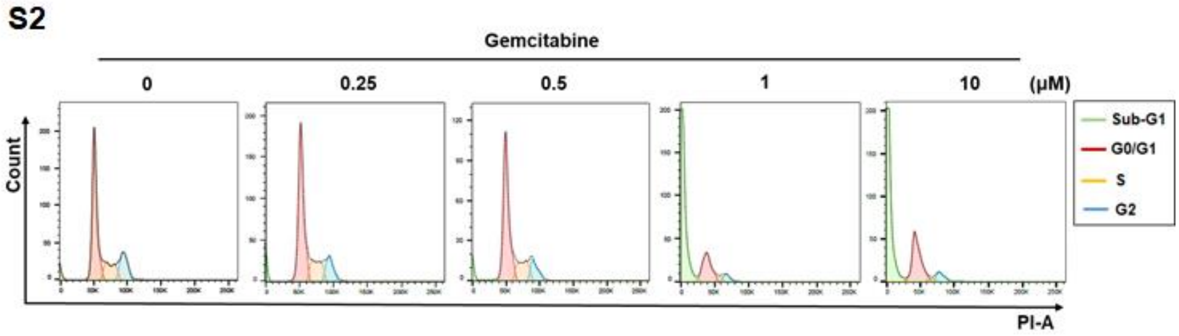

Figure S2: Determination of the optimal concentration of gemcitabine used for cell cycle analysis of Panc-1 cells

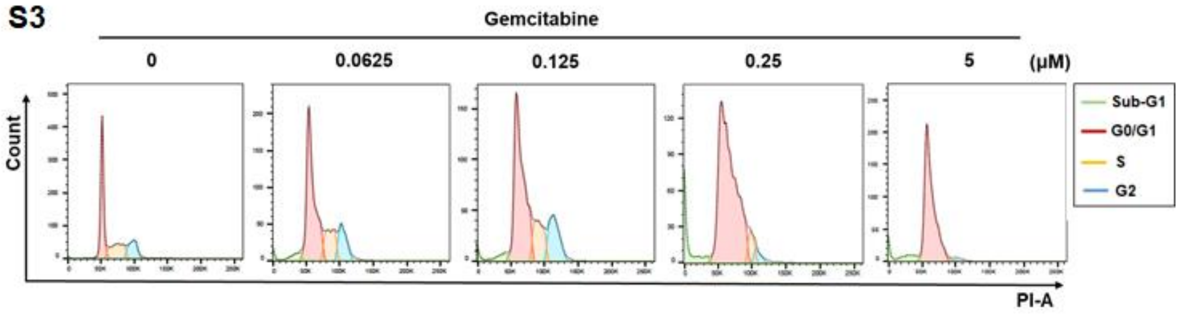

Figure S3: Determination of the optimal concentration of gemcitabine used for cell cycle analysis of AsPC-1 cells

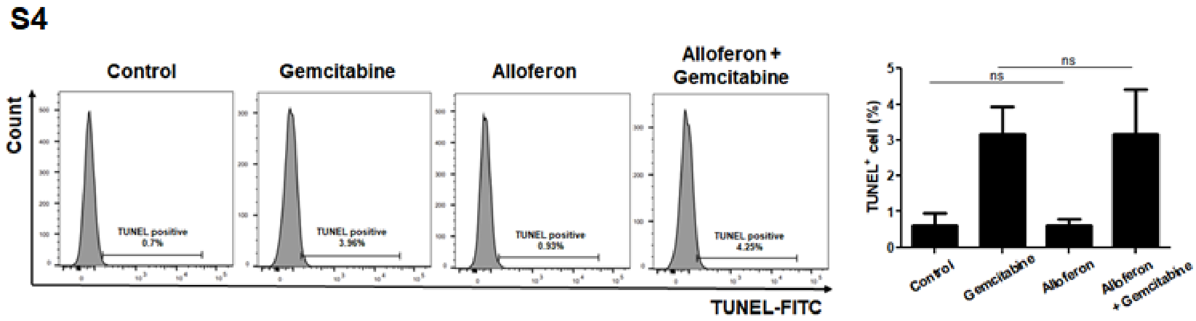

Figure S4: TUNEL positive cells of Panc-1 after exposure to alloferon and gemcitabine

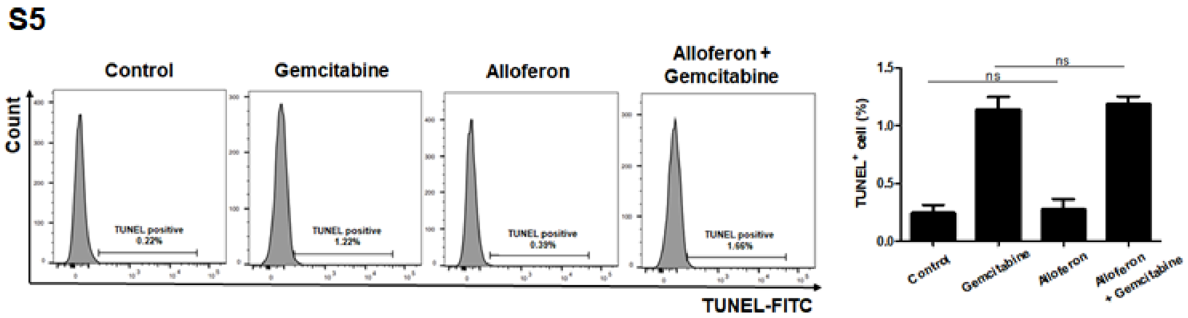

Figure S5: TUNEL positive cells of AsPC-1 after exposure to alloferon and gemcitabine
